# C3 molecular structural and histopathological analyses in a pediatric case of atypical hemolytic uremic syndrome with life-threatening gastrointestinal bleeding—a case report

**DOI:** 10.3389/fped.2025.1710286

**Published:** 2026-01-27

**Authors:** Takuji Enya, Kohei Miyazaki, Sakina Kuge, Yuichi Morimoto, Hiroki Kondou, Naoki Sakata, Kensuke Joh, China Nagano, Kandai Nozu, Nobutoshi Ito, Yoshiyuki Hakata, Keisuke Sugimoto, Masaaki Miyazawa

**Affiliations:** 1 Department of Pediatrics, Kindai University Faculty of Medicine, Osaka, Japan; 2 Department of Immunology, Kindai University Faculty of Medicine, Osaka, Japan; 3 Department of Pediatrics, Kindai University Nara Hospital, Nara, Japan; 4 Department of Pathology, Jikei University Hospital, Tokyo, Japan; 5 Department of Pediatrics, Kobe University Graduate School of Medicine, Kobe, Japan; 6 Medical Research Laboratory, Institute of Integrated Research, Institute of Science Tokyo, Tokyo, Japan; 7 Kindai University, Osaka, Japan

**Keywords:** atypical hemolytic uremic syndrome, C3 variant, C345C domain, gastrointestinal bleeding, refractory hypertension

## Abstract

Atypical hemolytic uremic syndrome (aHUS) is a rare, life-threatening disease characterized by uncontrolled complement activation and is associated with various genetic factors, including multiple variants at the gene locus encoding the complement component 3 (C3). However, only a few functional amino acid substitutions have been identified in C3. We report a pediatric case of aHUS presenting with refractory hypertension and massive gastrointestinal bleeding. Comprehensive histopathological, immunohistochemical, genetic, and molecular structural analyses were performed. Biopsy specimens were stained for renin and the membrane-attack complex of the complement system (C5b-9). Plasma levels of the Ba fragment of complement factor B were measured to evaluate the alternative pathway activation. Genomic DNA was obtained with consent and analyzed by targeted next-generation sequencing using a custom gene panel, followed by Sanger sequencing for variant confirmation. The possible effects of the identified amino acid substitution on the molecular structure of C3 were analyzed using computer-aided simulation with MODELLER and DoGSiteScorer. As a result, the juxtaglomerular apparatus was hyperplastic and intensely stained for renin. Endothelial cells of renal and intestinal blood vessels were positive for C5b-9. The plasma Ba level was elevated compared to the control level. The ileal and colonic mucosae were denuded and highly edematous, with epithelial cells undergoing regenerative and metaplastic changes in the active phase. Mucosal blood vessels contained intraluminal red cell fragments and neutrophils attached to swollen endothelial cells. However, the colonic mucosa showed near-normal histology after disease convalescence. Genetic analyses identified a single nucleotide C3 variant NM_000064.4(C3):c.4811T>C (p.Met1604Thr), resulting in an M_1604_T substitution in the functional C345C domain. Molecular structural analyses indicated that this amino acid substitution can cause the formation of a large cavity within the hydrophobic core of the C-terminal domain, possibly destabilizing the spherical structure of C3. Our study highlights that the M_1604_T substitution in the C3 C345C domain may drive the observed excessive complement activation, C5b-9 deposition on endothelial cells, and severe circulatory disturbances in the intestinal mucosa.

## Introduction

Atypical hemolytic uremic syndrome (aHUS) is a rare form of thrombotic microangiopathy (TMA) characterized by the triad of microangiopathic hemolytic anemia, thrombocytopenia, and acute kidney injury (AKI) in the absence of Shiga or Shiga-like toxin-producing enteropathogenic bacteria or severe reduction of a disintegrin and metalloproteinase with thrombospondin type 1 motif, member 13 (ADAMTS13) activity (1–3). Approximately 50% of aHUS patients progress to end-stage renal disease (ESRD) (2–5), but renal transplantation has a poor outcome due to frequent disease recurrence (2, 3). The pathogenesis of aHUS involves inherited or acquired defects in the regulation of complement activation via the alternative pathway (AP) (1–4). AP activation results in proteolytic cleavage of factor B, causing elevated plasma levels of its amino-terminal Ba fragment, a biomarker of AP stimulation (6). Between 60% and 70% of patients with aHUS possess variants in genes encoding the complement regulatory proteins, such as factor H, factor I, or membrane cofactor (CD46) (the *CFH*, *CFI*, and *MCP*, respectively), or in those encoding components of the AP C3-convertase C3 and factor B (the *C3* and *CFB*, respectively), genomic rearrangements between the *CFH* and related *CFHR* loci, or anti-complement factor H (CFH) antibodies (Ab) (7). Although C3 genetic alterations are reported less frequently than those of CFH (1–3), they are associated with more aggressive disease progression and a higher rate of ESRD or mortality (2, 3).

Extrarenal manifestations of aHUS can affect prognosis. Among these, gastrointestinal (GI) manifestations are frequently associated with homozygous defects in the *CFHR1* or *CFHR3* loci, resulting in the formation of anti-CFH Ab (2). However, in pediatric aHUS, GI complications are less common (1), mainly exhibiting vomiting and abdominal pain, and only 1.4% exhibit GI bleeding (8). Comorbidity of inflammatory bowel disease and aHUS has been reported in three pediatric cases, where gut inflammation might have served as a trigger of complement activation (9). Detailed histopathologic findings regarding GI manifestations of aHUS are rarely reported, except for a few adult or elderly cases (10–12), limiting our understanding of the pathophysiological relationship between complement activation, TMA, and intestinal damage, particularly in pediatric settings.

We previously encountered a pediatric aHUS patient with refractory hypertension and massive GI bleeding of up to 2,000 mL/day, beginning on hospital day 24 despite continuous hemodiafiltration (CHDF) and Eculizumab administration (13). In this report, we present detailed histopathological and immunohistochemical analyses of gut and kidney biopsy specimens, along with genetic and molecular structural investigations, to clarify the possible relationships between C3 genetic variation, complement activation, and the observed refractory hypertension and massive GI bleeding.

## Case presentation

A 3-year-old girl was admitted to Kindai University Hospital with hematuria, proteinuria, and generalized edema. On admission, she had proteinuria of 33.2 g/day, blood hemoglobin 6.7 g/dL (reference range: 11.0–14.2 g/dL), platelet count 66,000 /µL (180,000–580,000 /µL), urea nitrogen (BUN) 17.0 mg/dL (5.5–19.3 mg/dL), creatinine 0.57 mg/dL (0.2–0.38 mg/dL), total protein 4.0 g/dL (6.0–7.7 g/dL), albumin 2.0 g/dL (3.5–4.7 g/dL), and lactate dehydrogenase (LDH) 2,033 U/L (190–365 U/L). Schistocytes were observed in the peripheral blood, and the urine sediment contained >100 red cells per high-power field. The ADAMTS13 level was within the normal range (70%), and anti-dsDNA and anti-ssDNA auto-Ab were undetectable. Plasma C3 (78 mg/dL, reference range: 60–111 mg/dL) was within normal range, but C4 was decreased (10.0 mg/dL, reference range: 15–50 mg/dL). Anti-CFH Ab was undetectable. Stool cultures for enteropathogenic bacteria and detection of O157 LPS were both negative, and urine *Staphylococcus pneumoniae* antigen was undetectable. There was no family history of enterohemorrhagic *Escherichia coli* (EHEC) infection, and the patient had no remarkable past medical history. Based on these findings, aHUS was diagnosed. Plasma infusion (PI) was performed on hospital day 2. However, as BUN increased to 36 mg/dL, creatinine 0.94 mg/dL, and LDH 2,841 U/L, 600 mg of Eculizumab was administered on day 4 based on the patient's body weight of 14.3 kg on admission. On day 8, the patient was transferred to the intensive care unit (ICU) due to clinical aggravation, with blood pressure 220/112 mmHg, BUN 60 mg/dL, creatinine 2.23 mg/dL, and pulmonary edema. Plasma exchange (PE) was performed with CHDF under mechanical ventilation. After five daily PE sessions through the 12th day, 600 mg of Eculizumab was readministered as the initiating dose, followed by maintenance doses of 300 mg every two weeks starting on the 19th day. Negative results of the secondary stool culture and O157 LPS detection on day 21 further excluded EHEC infection. Her kidney function gradually improved, and CHDF was discontinued on day 23. Nevertheless, bloody diarrhea began on the 20th day and increased to more than 2.0 L/day, which continued from day 24 onward, requiring frequent red cell transfusion and albumin injection. Informed consent was obtained from the patient's parents for the colonofiberscopy with ileal and sigmoid colon biopsy, which was performed on day 30. No pseudomembrane formation was observed, and stool *Clostridioides difficile* (CD) toxin and a blood C7-HRP test for cytomegalovirus (CMV) pp65 were both negative. Upon initiation of octreotide acetate drip infusion on day 37 and its increase to 0.5 µg/kg/h on day 40, bloody diarrhea stopped. However, her malignant hypertension (MHT) became refractory to treatment with furosemide, carperitide, a large intravenous dose of nicardipine chloride, and nitroglycerine. Moreover, high levels of plasma renin activity (62 ng/mL/h, reference range: 1.5–3.5 ng/mL/h) and serum aldosterone (484 pg/mL, reference range: 49–179 pg/mL) were detected on day 47. Therefore, renin–angiotensin system (RAS) inhibitors for refractory hypertension, along with the above biweekly Eculizumab administration, were adopted, which proved effective. In particular, Enalapril (2.5 mg/day) was initiated on day 48, and Candesartan (2 mg/day) was added on day 54. She was discharged from the ICU on day 56. Candesartan was continued for approximately 3 months, whereas Enalapril has been maintained long-term. A follow-up colonofiberscopy was performed to confirm treatment efficacy on day 102, followed by a renal biopsy on day 114, with written informed consent from the patient's parents. She was discharged from the hospital on day 137. Ravulizumab was regularly administered as part of outpatient care. Her kidney function recovered to chronic kidney disease stage 2, and no recurrence has been observed during more than 5 years of follow-up.

**Figure 1:**
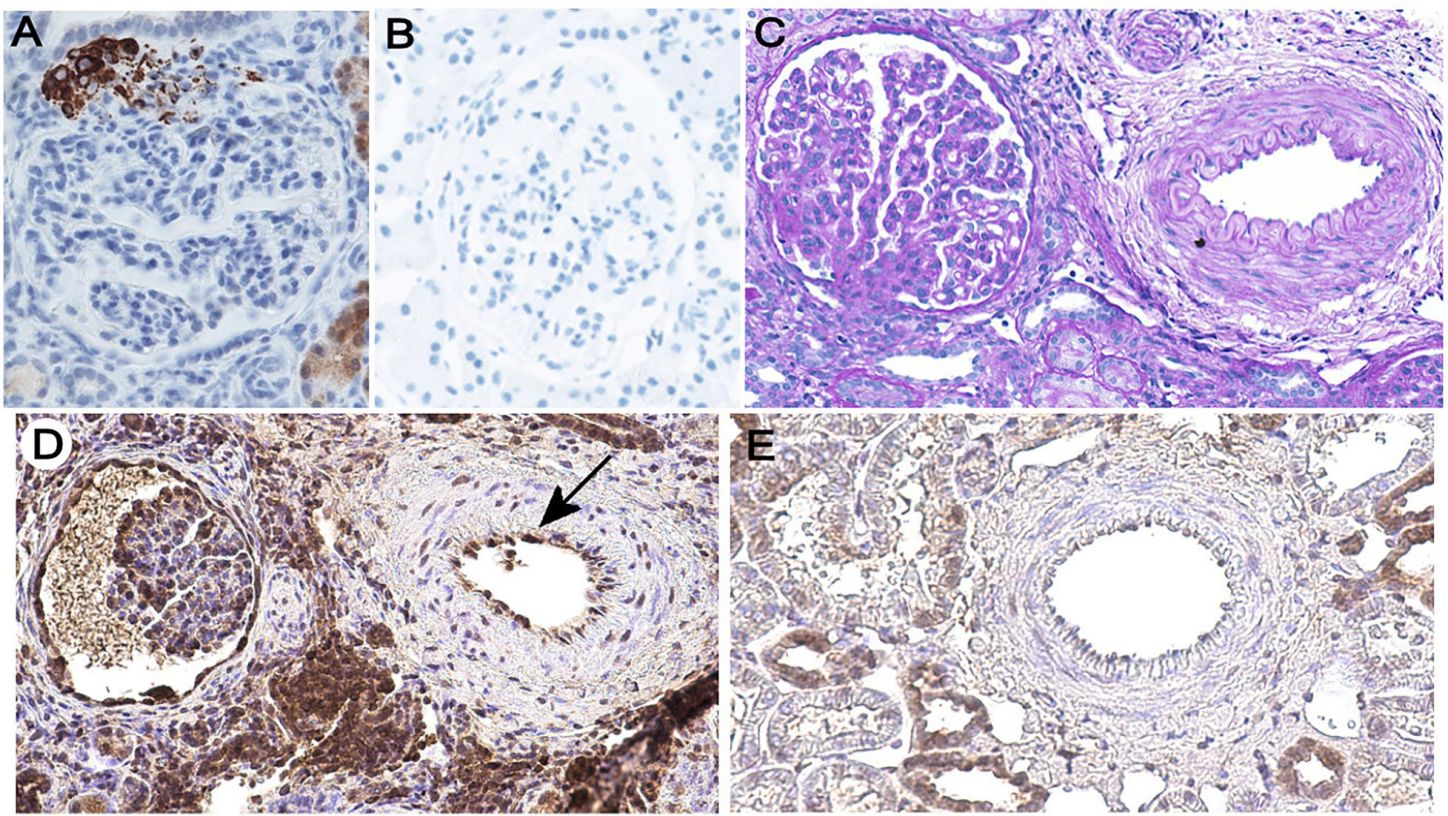
Immunohistochemical analyses of the kidney specimens. **(A)** Immunohistochemical staining for renin with the anti-renin monoclonal Ab showing intense tinction of the JGA (×400). Discovery Ultra Autostainer (Ventana Medical Systems) with an I-VIEW 3,3′-diaminobenzidine (DAB) kit was used for renin staining. **(B)** A control section incubated with normal mouse IgG1 instead of the anti-renin primary Ab showed no staining (×400). **(C)** PAS staining shows thickening of the renal arteriolar smooth muscle layer (×200). **(D)** Immunohistochemical staining for C5b-9 shows its deposition on the renal arteriolar endothelial cells (arrow) (×200). For C5b-9 detection, the sections were treated with 0.01 M citric acid at 121°C for 10 min for antigen retrieval, and endogenous peroxidase was blocked for 10 min with 0.3% hydrogen peroxide in methanol. After Fc receptor blocking with monoclonal Ab 2.4G2 (Tonbo biosciences) for 60 min, the sections were incubated with anti-C5b-9 polyclonal Ab (Abcam 55811) diluted at 1:1,000 at 4°C for 24 h. After washing, the specimens were incubated with the biotinylated anti-rabbit IgG secondary Ab (Cappel 39724) for 60 min and reacted with the VECTASTAIN Elite ABC reagent (Vector) for 30 min, after which the DAB substrate was added. **(E)** A control section obtained from a pediatric patient pathologically diagnosed with minimal change disease is negative for endothelial staining for C5b-9 (×200).

Immunohistochemical analyses of the formalin-fixed and paraffin-embedded renal biopsy specimens with an anti-renin mouse monoclonal Ab (clone 411507, R&D Systems) revealed intense staining of the hyperplastic juxtaglomerular apparatus (JGA) (Figure 1A), consistent with the high plasma renin activity. Control sections incubated with unimmunized mouse IgG1 showed no staining (Figure 1B). Immunofluorescence microscopy for immunoglobulin, C3, and C4 depositions was negative (not shown). Periodic acid Schiff (PAS) staining demonstrated mesangiolysis, diffuse mesangial proliferation, thickening and double contours of glomerular capillary walls, and glomerular sclerosis, all consistent with TMA. In addition, thickened vascular walls and narrowed lumen in renal arterioles reflected sustained hypertension (Figure 1C). Endothelial cells of thickened renal arterioles were positive for C5b-9 by immunostaining with an anti-C5b-9 polyclonal Ab (Abcam 55811) (Figure 1D, arrow). Renal biopsy specimens obtained from a patient with minimal change disease used as controls showed no staining of endothelial cells (Figure 1E).

**Figure 2:**
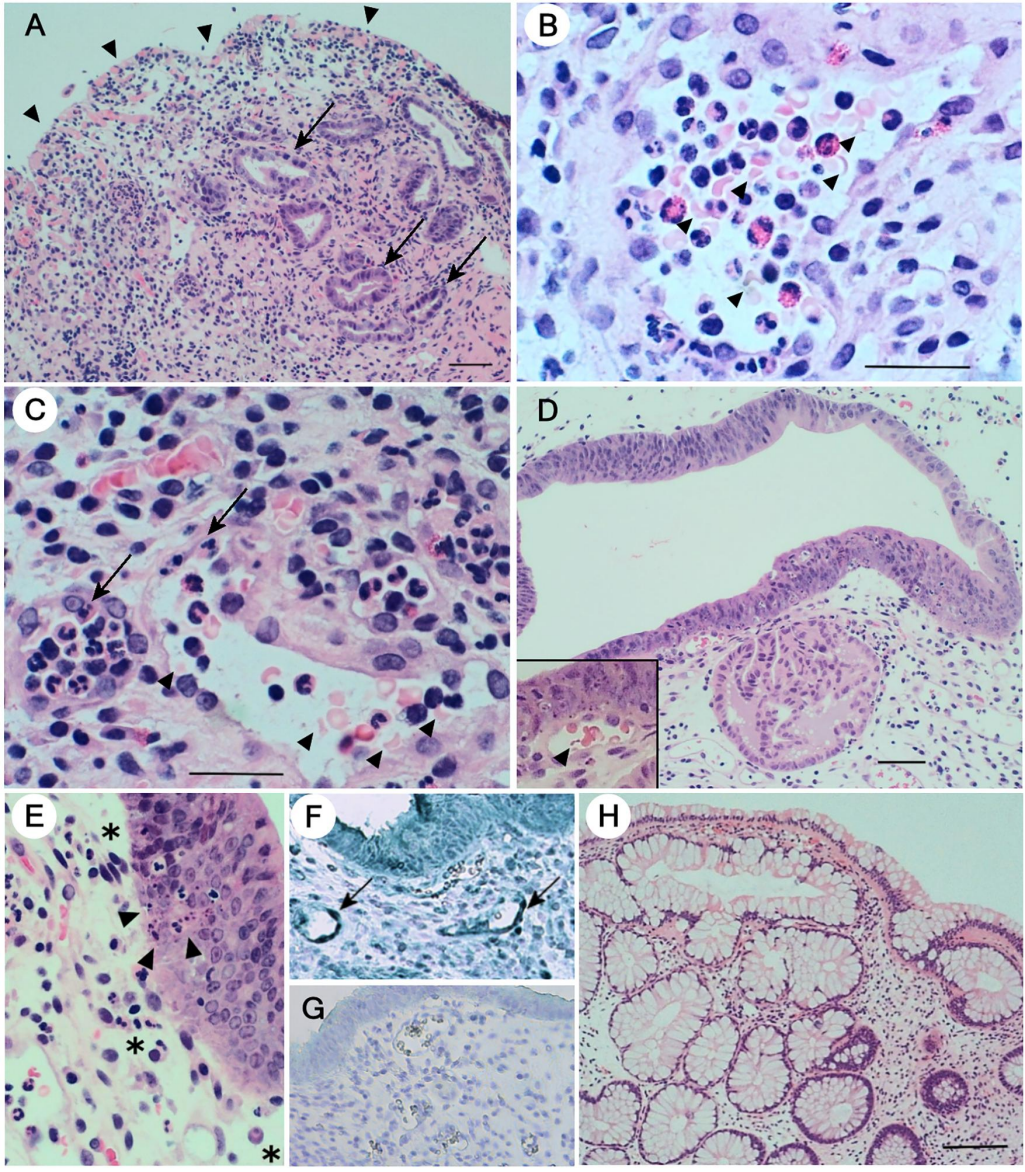
Histopathological and immunohistochemical features of gut biopsy specimens. **(A)** Hematoxylin and eosin (HE)-stained section of the patient's terminal ileum. Surface denudation (arrowheads), loss of glands, and edema of the lamina propria with diverse inflammatory cell infiltration are all consistent with histopathologic manifestations of intestinal TMA (30). Bar = 50 µm. **(B,C)** A higher magnification of the ileum (Bar = 25 µm) demonstrating intraluminal red cell fragments (arrowheads) in dilated blood vessels with swollen endothelial cells. Attachment of neutrophils on endothelial cells is evident (arrows). **(D)** HE-stained section of the patient's sigmoid colon (Bar = 50 µm). Edema of the lamina propria and peculiar metaplastic changes of the epithelium are evident. Intraluminal red cell fragments are also observed in dilated blood vessels (arrowhead in the inset). **(E)** A higher magnification of the sigmoid colon shows edema in the lamina propria (*) and numerous foci of apoptotic cells (arrowheads) in the stratified epithelial layer. **(F)** Immunohistochemical staining for C5b-9 of the same sigmoid colon. Endothelial cells of the dilated blood vessels are positive (arrows) (×400). **(G)** Staining of the same specimen with control rabbit IgG showed no positive signals (×400). For the detection of C5b-9 in sigmoid colon specimens, the sections were reacted with anti-rabbit Novolink polymer (Leica RE7112) at room temperature (RT) for 30 min, followed by Histogreen staining (Linaris, E109). **(H)** The sigmoid colon specimen obtained on day 102 of hospitalization reveals almost normal morphology with Goblet cell-containing tubular structures. Bar = 100 µm.

Biopsy specimens from the terminal ileum obtained during colonoscopy on hospital day 30 revealed total denudation, loss of glands, and massive edema of the lamina propria with diverse inflammatory cell infiltration, including lymphocytes and plasma cells (Figure 2A, arrowheads). Remaining tubular epithelial cells were regenerative and metaplastic, exhibiting anisonucleosis (arrows). At higher magnification (Figures 2B,C), dilated small blood vessels with swollen endothelial cells contained numerous red cell fragments (arrowheads), consistent with clinical TMA, and showed neutrophil attachment to endothelial cells (arrows), indicative of possible complement-mediated cell adhesion. Sigmoid colon specimens demonstrated massive edema in the lamina propria (Figure 2D), with regenerative epithelial cells exhibiting extensive stratification and peculiar metaplasia. Dilated blood vessels contained red cell fragments (arrowhead in the inset). Higher magnification revealed edema (*) and numerous foci of aggregated apoptotic epithelial cells (arrowheads). Endothelial cells of dilated capillaries and small arterioles were positive for C5b-9 (Figure 2F, arrows), whereas using control rabbit IgG resulted in no significant staining (Figure 2G). Sigmoid colon specimens obtained on day 102 restored almost normal mucosal structures (Figure 2H) with reappearance of Goblet cells.

**Figure 3:**
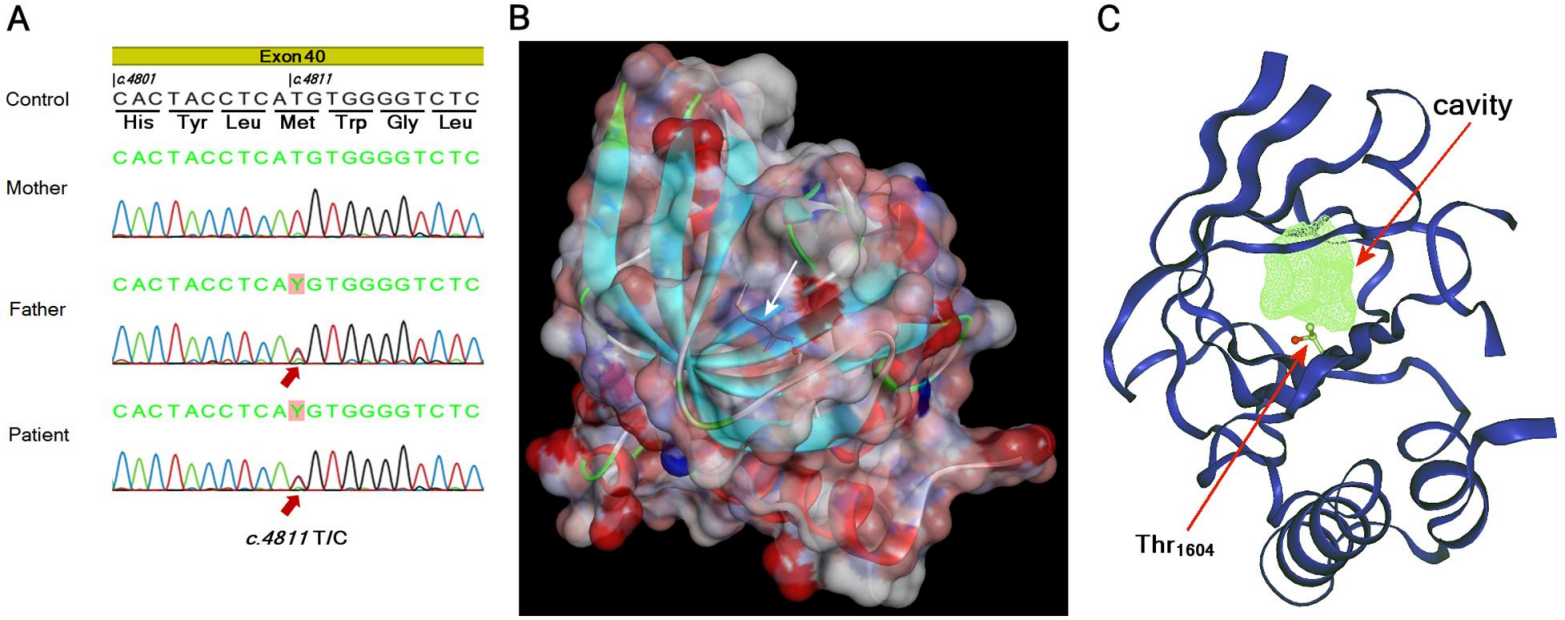
C3 gene sequencing and molecular structural analyses. **(A)** Targeted next-generation sequencing identified a heterozygous NM_000064.4(C3):c.4811T>C (p.Met1604Thr) variant, which was confirmed by Sanger sequencing. **(B)** A structural model of the entire C345C domain was rendered with the MODELLER software (16) using a crystal structure of the CTC domain of C3/C3b (PDB entry 6S0B) (17). The M at position 1604 (white arrow) is buried in the hydrophobic core surrounded by overlapping β-sheets. **(C)** Internal cavities were searched for wild-type and variant proteins using DoGSiteScorer (18), available via a website (https://proteins.plus/). DoGSiteScorer indicated the formation of an approximately 100 Å^3^ cavity (green mesh) near position 1604 when this residue is a T (shown in ball and stick). The cavity was not detected in the wild-type protein.

The patient's peripheral blood was collected in EDTA-containing tubes on day 1 before PI, and the plasma was stored at −80°C until assayed. Plasma Ba levels were retrospectively determined using an ELISA kit (QuidelOrtho Corporation, San Diego, CA, USA) and were 2,546 ng/mL (reference range: 226–2,153 ng/mL; median: 461.2 ng/mL) during the active phase. The patient's plasma Ba level during convalescence was 702 ng/mL. Genomic DNA was extracted from peripheral blood using QuickGene-Auto 12S (Kurabo Industries Ltd., Osaka, Japan). Targeted next-generation sequencing was performed using a custom-designed gene panel for inherited kidney diseases (Supplementary Table S1) as described previously (14). The panel included complement-related genes implicated in aHUS: *C3*, *CFH*, *CFI*, *CD46* (*MCP*), *CFB*, *THBD*, *CFHR1*, and *DGKE* (15). Library preparation was performed using the SureSelect XT2 custom capture system (0.5–2.9 Mb; Agilent Technologies, Santa Clara, CA, USA), followed by paired-end sequencing on the Illumina MiSeq platform. Data processing and variant calling were performed with SureCall 3.0. The identified NM_000064.4(C3):c.4811T>C (p.Met1604Thr) variant was confirmed by Sanger sequencing using the following primers: forward, 5ʹ-GGTGCACTTGCAAACCAG-3ʹ; reverse, 5ʹ-GGCGTGACAATGGTGTGG-3ʹ (Figure 3A). Given that alterations in the *CFH–CFHR1* region may contribute to aHUS development, copy number variation (CNV) analysis was performed using read-depth-based CNV calling implemented in SureCall. This analysis demonstrated no evidence of pathogenic deletions or duplications in this region. Consequently, the patient's *C3* gene locus harbored a heterozygous missense variant, with methionine (M) at position 1604 replaced by threonine (T) in the C345C domain. Segregation analyses confirmed that the patient's father, who had no medical history of aHUS, was also heterozygous for the same variant (Figure 3A). We confirmed that no other proximal relatives had a history of renal diseases.

The identified amino acid substitution was predicted, in preliminary analyses using JPred 4 (https://www.compbio.dundee.ac.uk/jpred/), to alter the secondary structure of the segment containing nearby tryptophan (W) and phenylalanine (F) residues, thereby potentially destabilizing the entire domain. Therefore, molecular modeling of the M_1604_T variant was performed with MODELLER (16) using the crystal structure of the C3/C3b CTC domain (PDB entry 6S0B) as a template (17). The results indicated that this M at position 1604 is completely buried within the protein (Figure 3B, white arrow). Substitution with a less bulky T was predicted to create a cavity inside the protein. Indeed, the analysis with DoGSiteScorer (18) identified a cavity of approximately 100 Å^3^ near position 1604 in the variant but not in the wild-type protein (Figure 3C). The cavity appeared to be highly hydrophobic, surrounded by residues including M_1563_, F_1584_, isoleucine (I)_1622_, and valine (V)_1682_, and was very unlikely to be filled by water molecules in solution.

## Discussion

Approximately 40%–60% of aHUS patients are reported to possess identifiable variants in genes encoding complement components of the AP (1–3). These variants lead either to loss of function in regulatory factors, including factor H, factor I, and CD46, or to gain of function in effector proteins, such as factor B, C3, or thrombomodulin (5, 19, 20). In Japan, pathogenic variants in the *C3* gene account for ≥30% of aHUS cases, with the amino acid substitutions implicated including S_179_P, S_182_P, P_214_S, R_425_C, V_555_I, R_1042_L, K_1105_Q, I_1157_T, and E_1160_K (21, 22). Reduced C3b degradation resulting from decreased binding to factor H or CD46 causes excessive complement activation (2, 23). The C345C domain of C3 is hypothesized to function as a binding site for factor B, which is required for C3 convertase formation (24–26). This domain undergoes large-scale structural rearrangements upon proteolytic activation of C3 and is present in the C3b fragment (27, 28). Furthermore, the same domain is implicated in C5 convertase activity (26). In the present study, we identified a heterozygous missense variant in the *C3* gene in an aHUS patient resulting in M_1604_T substitution in the C345C domain. Although the same variant in the *C3* gene was previously reported in a single case, the possible pathogenetic significance of the M_1604_T substitution has not been demonstrated (29). In the present study, molecular structural analyses indicated the M_1604_T substitution's possible functional significance as cavity formation within the hydrophobic core of the C345C domain. Unfilled cavities within proteins are known to destabilize them, and the cavity found in the indicated hydrophobic core is likely to cause the same effect. There are possibilities that the rearrangement of side chains in nearby amino acid residues and/or changes in the main chain conformation may cancel the cavity formation. However, such structural adaptation may not be able to cancel the destabilizing effect completely. This structural instability may result in accelerated proteolytic cleavage of C3 and interactions with factor B, contributing to the observed higher Ba levels, deposition of C5b-9 on endothelial cells, and putative endothelial cell damage with neutrophil, and conceivably thrombocyte, attachment.

At admission, plasma C3 concentration in our patient was within the reference range. In aHUS patients with *C3* or *CFB* variants, serum C3 levels are below the normal range in >70% of cases (30, 31). However, serum C3 levels in aHUS patients with *C3* variants span a wide range and do not necessarily cluster below the reference range cutoff (30), limiting their prognostic significance (31). In fact, up to 30% of aHUS patients with pathogenic *C3* or *CFB* variants have normal serum C3 levels (30, 31). Thus, normal C3 levels do not exclude aHUS (32). The patient's plasma Ba level was only mildly elevated. Plasma samples for Ba quantification in the present study were first obtained on hospital day 1, prior to PI, and stored at −80°C. As ELISA assays were performed retrospectively, it is possible that Ba immunoreactivity was affected during storage. However, it is also known that plasma Ba values are much lower than serum values and tend to accumulate at lower levels (33). Our patient's plasma Ba levels were higher than the upper limit of the reference range during the active phase and decreased during convalescence, suggesting a presumable correlation between Ba levels and disease activity. The demonstration of C5b-9 deposition on vascular endothelial cells unequivocally indicates complement activation as the cause of TMA in this case.

The patient's father was also heterozygous for the same genetic variant but did not present any relevant symptoms or family history. This is not surprising, as the penetrance of pathogenic C3 variants in aHUS is approximately 20% (29), and a triggering event is often required for disease manifestation (1, 2). Although prodromal diarrhea is common among aHUS patients (1), our patient lacked such a history. Instead, she experienced a common cold-like acute upper respiratory tract infection one month prior to the initial development of pretibial edema. Further, bloody diarrhea developed on hospital day 20 and worsened on day 24, indicating that the GI manifestation was a result, rather than a trigger, of aHUS-related TMA. Histopathological findings—including surface denudation, loss of glands, intraluminal red cell fragments, and neutrophil attachment to swollen endothelial cells in dilated blood vessels, along with C5b-9 deposition on endothelial cells and massive edema of the lamina propria—are consistent with previously reported histologic manifestations of intestinal TMA (34), indicating TMA-associated mucosal circulatory disturbances as a cause of bloody diarrhea. The absence of pseudomembrane formation on colonofiberscopy, negative results of stool CD toxin and blood C7-HRP tests, and normal levels of α1-antitrypsin exclude CD- or CMV-induced enteritis and protein-losing enteropathy as alternative causes of bloody diarrhea. However, a relatively low C4 level in the absence of anti-CFH Ab may indicate complement activation via the mannose pathway, associated with an unidentified microbial infection in the initial phase of the disease development. The anti-secretory effects of octreotide acetate successfully suppressed gastrointestinal bleeding. In addition to supportive management, a combination of PE and Eculizumab was effective, resulting in extensive regeneration of the intestinal epithelium, as confirmed by colonofiberscopy on day 102.

Hypertension is a common complication of aHUS, and calcium channel blockers are used as first-line therapy in Japan. However, aHUS-associated hypertension is often resistant to these agents. Anti-C5 Ab Eculizumab is reported to be effective in treating aHUS-associated hypertension (35–37), while some cases are refractory (35). The pathophysiology of MHT is complex, but the local activation of the RAS is considered important, leading to a vicious cycle of progressive kidney damage and intractable hypertension (38). In fact, renin-positive JGA hyperplasia associated with high plasma renin activity and aldosterone levels was observed in our patient, justifying the use of RAS inhibitors, which resulted in a good response.

These findings are consistent with the hypothesis that excessive C3 activation, associated with the detected C-terminal domain amino acid substitution and resultant molecular instability, led to uncontrolled C5b-9 deposition on vascular endothelial cells. The resultant TMA underlies the development of severe extrarenal manifestations observed in our case, including MHT and GI bleeding.

## Limitations

Although analyses of CNV in the *CFH-CFHR1* region were performed and anti-CFH Ab was not detected, we did not directly assess the possible complex genomic rearrangements involving the region harboring the *CFHR*s in this study. The effect of the M_1604_T substitution on the stability of the C3 molecule was predicted by our computational modeling. However, possible changes in the rate of C3 cleavage and binding to factor B were not directly examined. Further studies, including recombinant protein expression and surface plasmon resonance analyses, are required to directly demonstrate the effect of the M_1604_T substitution on the putatively altered functionality of the variant C3 protein. The C3 variant NM_000064.4(C3):c.4811T > C (p.Met1604Thr) has been classified as a variant of uncertain significance (29) based on the American College of Medical Genetics and Genomics and the Association for Molecular Pathology (ACMG/AMP) standards and guidelines for the interpretation of sequence variants (39). While the identified missense variant results in the M_1604_T amino acid substitution within the predicted functional domain of the C3 molecule (24–28), several missense variants within the coding sequence of this domain—NM_000064.4(C3):c.4535G>A (p.Arg1512His), NM_000064.4(C3):c.4759C>T (p.Pro1587Ser), and NM_000064.4(C3):c.4855A>C (p.Ser1619Arg)—have been classified benign or likely benign for the development of aHUS (ClinVar Variation/condition reports RCV000264234.5, RCV000261966.5, and RCV000395497.7 and RCV005863100.1, respectively). On the other hand, the allele frequency of the NM_000064.4(C3):c.4811T>C variant is extremely low at 0.0004234 for the East Asian genetic ancestry group (0.00001363 for the total ancestry groups), with no homozygosity found (https://gnomad.broadinstitute.org/variant/19-6678191-A-G?dataset=gnomad_r4). Computational evidence—both SIFT (https://sift.bii.a-star.edu.sg/) and MutationTaster2025 (https://www.genecascade.org/MutationTaster2025/)—listed in the ACMG/AMP standards and guidelines, predicted the M_1604_T substitution to be deleterious (SIFT Score 0 and Tree vote 77/23, respectively), in addition to our computational analyses predicting molecular instability. However, with moderate and supporting evidence of pathogenicity (PM2 and PP3), this variant remains classified as of uncertain significance.

## Conclusions

We have identified that an aHUS-associated C3 variant with M_1604_T substitution in the C345C domain may cause molecular instability. Since the C345C domain contributes to the binding between C3b and Bb, such functional alterations may lead to excessive proteolytic cleavage of the substrate by the AP C3 convertase, resulting in the deposition of the membrane-attack complex on endothelial cells and the onset of life-threatening manifestations, including refractory hypertension and massive GI bleeding. However, timely monitoring of the possible pathophysiological factors and vigorous intervention can reverse the process of MHT and severe GI damage, ultimately saving the patient's life.

## Data Availability

The original contributions presented in the study are included in the article/Supplementary Material; further inquiries can be directed to the corresponding authors.
