# Supplementary material for: C3 molecular structural and histopathological analyses in a pediatric case of atypical hemolytic uremic syndrome with life-threatening gastrointestinal bleeding—a case report
Source: Front Pediatr. 2026 Jan 27;13:1710286. doi: 10.3389/fped.2025.1710286 (PMC12886399; doi:10.3389/fped.2025.1710286)
Supplement: Supplementary file 1 [file Table1.docx]

**Supplementary Table 1.** List of genes included in the targeted sequencing panel.

| *ACTN4* | *ADAMTS13* | *ADCK4* | *ANKFY1* | *ANLN* | *AQP2* | *ARHGAP24* | *ARHGDIA* | *ATP6V0A4* |
| --- | --- | --- | --- | --- | --- | --- | --- | --- |
| *ATP6V1B1* | *AVIL* | *AVP* | *AVPR2* | *BCS1L* | *BSND* | *C3* | *CA2* | *CASR* |
| *CD2AP* | *CD46* | *CDK20* | *CFB* | *CFH* | *CFHR1* | *CFI* | *CFTR* | *CLCN5* |
| *CLCNKA* | *CLCNKB* | *CLDN10* | *CLDN16* | *CLDN19* | *CNNM2* | *COL4A1* | *COL4A3* | *COL4A4* |
| *COL4A5* | *COQ2* | *COQ6* | *CRB2* | *CTNS* | *CUBN* | *CUL3* | *DGKE* | *DLC1* |
| *EGF* | *EHHADH* | *EMP2* | *EYA1* | *FAT1* | *FN1* | *FXYD2* | *GAPVD1* | *GLA* |
| *GON7* | *HNF1B* | *INF2* | *ITGA3* | *ITGB4* | *ITSN1* | *ITSN2* | *KANK1* | *KANK2* |
| *KANK4* | *KCNA1* | *KCNJ1* | *KCNJ10* | *KIRREL1* | *KLHL3* | *LAGE3* | *LAMA5* | *LAMB2* |
| *LMNA* | *LMX1B* | *MAGED2* | *MAGI2* | *MUC1* | *MYH9* | *MYO1E* | *NPHS1* | *NPHS2* |
| *NR3C2* | *NUP107* | *NUP133* | *NUP160* | *NUP205* | *NUP85* | *NUP93* | *OCRL* | *OSGEP* |
| *PAX2* | *PCBD1* | *PDSS2* | *PLCE1* | *PTPRO* | *REN* | *SCARB2* | *SCNN1A* | *SCNN1B* |
| *SCNN1G* | *SEC61A1* | *SGPL1* | *SIX2* | *SLC12A1* | *SLC12A3* | *SLC26A3* | *SLC2A2* | *SLC34A1* |
| *SLC4A1* | *SLC4A4* | *SMARCAL1* | *THBD* | *TNS2* | *TP53RK* | *TPRKB* | *TRPC6* | *TRPM6* |
| *TTC21B* | *UMOD* | *WDR4* | *WDR73* | *WNK1* | *WNK4* | *WT1* | *XPO5* |  |
